# Defrosting the Digital Library: Bibliographic Tools for the Next Generation Web

**DOI:** 10.1371/journal.pcbi.1000204

**Published:** 2008-10-31

**Authors:** Duncan Hull, Steve R. Pettifer, Douglas B. Kell

**Affiliations:** 1 School of Chemistry, The University of Manchester, Manchester, United Kingdom; 2 The Manchester Interdisciplinary Biocentre, The University of Manchester, Manchester, United Kingdom; 3 School of Computer Science, The University of Manchester, Manchester, United Kingdom; National Center for Biotechnology Information (NCBI), United States of America

## Abstract

Many scientists now manage the bulk of their bibliographic information electronically, thereby organizing their publications and citation material from digital libraries. However, a library has been described as “thought in cold storage,” and unfortunately many digital libraries can be cold, impersonal, isolated, and inaccessible places. In this Review, we discuss the current chilly state of digital libraries for the computational biologist, including PubMed, IEEE Xplore, the ACM digital library, ISI Web of Knowledge, Scopus, Citeseer, arXiv, DBLP, and Google Scholar. We illustrate the current process of using these libraries with a typical workflow, and highlight problems with managing data and metadata using URIs. We then examine a range of new applications such as Zotero, Mendeley, Mekentosj Papers, MyNCBI, CiteULike, Connotea, and HubMed that exploit the Web to make these digital libraries more personal, sociable, integrated, and accessible places. We conclude with how these applications may begin to help achieve a digital defrost, and discuss some of the issues that will help or hinder this in terms of making libraries on the Web warmer places in the future, becoming resources that are considerably more useful to both humans and machines.

*“The apathy of the academic, scientific, and information communities coupled with the indifference or even active hostility…of many publishers renders literature-data-driven science still inaccessible.”* – Peter Murray-Rust [1]

## Introduction

The term *digital library* [2]–[4] denotes a collection of literature and its attendant metadata (data about data) stored electronically. According to Herbert Samuel, a library is “thought in cold storage” [5], and unfortunately digital libraries can be cold, isolated, impersonal places that are inaccessible to both machines and people. Many scientists now organize their knowledge of the literature using some kind of computerized reference management system (BibTeX, EndNote, Reference Manager, RefWorks, etc.), and store their own digital libraries of full publications as PDF files. However, getting hold of both the data (the actual publication) and the metadata for any given publication can be problematic because they are often frozen in the isolated and icy deposits of scientific publishing. Because each library and publisher has different ways of identifying and describing their metadata, using digital libraries (either manually or automatically) is much more complicated than it needs to be [6], and with papers in the life sciences alone (at Medline) being published at the rate of approximately two per minute [7], only computerized analyses can hope to be reasonably comprehensive. What then, are these digital libraries, and what services do they provide?

As far as computational Biologists are concerned, and for the purposes of this Review, we shall define a digital library more broadly as a database of scientific and technical articles, conference publications, and books that can be searched and browsed using a Web browser. As of early 2008, there is a wide range of these digital libraries, but no single source covering all information (in part because of the cost, given that there are some 25,000 peer-reviewed journals publishing some 2.5 million articles per year [8]). Each library is isolated, balkanized, and has only partial coverage of the entire literature. This contrasts with the historically pre-eminent library of Alexandria whose great strength was that it brought together all the useful literature then available to a single location. Like Alexandria, most digital libraries are currently *read-only*, allowing users to search and browse information, but not to *write* new information nor add personal knowledge. Other digital libraries are in danger of becoming *write-only* “data-tombs” [9], where data are deposited but will probably never be accessed again. Indeed, the literature itself is now so vast that most scientists choose to access only a fraction of it [10], at potentially considerable intellectual loss [11] (see also [12]).

Digital libraries provide electronic access to documents, sometimes just to their abstracts and sometimes to the full text of the publication. Presently, the number of abstracts considerably exceeds the number of full-text papers, but with the emergence of Open Access initiatives (e.g., [13]–[16]), Institutional Repositories (e.g., [17]–[20]), and the like, this is set to change considerably. This is very important, as much additional information exists in full papers that is not seen in abstracts, and, in addition, full papers that are available electronically are likely to be much more widely read and cited [21]–[23]. The format of the full text of such documents can vary significantly among publishers. Such formats can be described using a Document Type Definition (DTD), e.g., that provided by the (U.S.) National Library of Medicine [16],[24], and, since not all publishers (especially those of non-biomedical material) conform to the NLM DTD, this can considerably affect the types of analysis that can be done on such documents.

In a similar vein, there is not yet a recognized (universal) standard for describing the metadata (see Table 1), although some (discussed below) such as the Dublin Core are becoming widely used.

**Table 1:** A summary of some of the digital libraries described in this Review.

Name	Domain	Size	Style of Metadata	Persistent Inbound Links?	Persistent Outbound Links?	Full Text?	Access
ACM Digital Library http://portal.acm.org	Computer science	>54,000 articles	BibTeX, EndNote	Yes, see ACM section in text	Not applicable	For subscribers	Metadata and abstract free, full paper for subscribers only
IEEE Xplore http://ieeexplore.ieee.org	Computer science	Unknown	EndNote, Procite, Refman	Yes, see Xplore section in text	Not applicable	For subscribers	Metadata and abstract free, full paper for subscribers only
DBLPDBLP http://dblp.uni-trier.de	Mostly computer science	>900,000 articles	BibTeX	Yes, see dblp section in text	Various, including DOIs	Links to publisher DOIs	Metadata free
Pubmed http://pubmed.gov	Life sciences and biomedicine	>17,000,000 articles	XML, NLM, DTD	Yes, see PubMed section in text	LinkOut and links to publisher sites	Links to publisher DOIs	Metadata and abstract free
PubmedCentral http://pubmedcentral.gov	Life sciences and biomedicine	>750,000	XML, Dublin Core, RDF	Yes, see text	Not applicable	Yes	Free access to data and metadata
Web of Knowledge http://apps.isiknowledge.com	Broad scientific coverage	>15,000,000	BibTeX, EndNote, Refman, Procite	No, see WoK section in text	Links to publisher sites	Links to publisher DOIs	Subscription only
Scopus http://www.scopus.com	Broad scientific coverage	>33,000,000	RefWorks, EndNote, Refman, Procite	Yes, see Scopus section in text	Links to publisher sites	Links to publisher DOIs	Subscription only
Citeseer http://citeseer.ist.psu.edu	Broad coverage	>760,000	BibTeX	Yes, see Citeseer section in text	Local cache and links to self-archived papers	Yes	Free access
Google Scholar http://scholar.google.com	Broad coverage	Not published	Nothing very exportable, html only	Yes, see Google Scholar section in text	Direct links to publishers and self-archived grey literature	Yes (includes grey literature and self-archived)	Free access
arXiv http://www.arxiv.org/	Mainly physical sciences	>44,000	BibTeX,	Yes, see section on arXiv in text	Links to self-archived material in some PDFs	Yes	Free access

Note that this table summary does not cover all the minutiae of licensing issues.

Since all of these libraries are available on the Web, increasing numbers of tools for managing digital libraries are also Web-based. They rely on Uniform Resource Identifiers (URIs [25] or “links”) to identify, name, and locate resources such as publications and their authors. By using simple URIs, standard Web browser technology, and the emerging methods of the next generation Web or “Web 2.0” [26], it has become possible for digital libraries to become not just *read-only* or *write-only*, but both *read–write*. These applications allow users to add personal metadata, notes, and keywords (simple labels or “tags” [27],[28]) to help manage, navigate, and share their personal collections. This small but significant change is helping to improve digital libraries in three main ways: personalization, socialization, and integration.

The focus of this Review is largely about searching and organizing literature data together with their metadata. For reasons of space, we do not consider in any detail issues surrounding Open Access (e.g., [13],[29]), nor structured digital abstracts [30],[31] (note the recent initiative in FEBS Letters [32]–[34] and the RSC's Project Prospect for whole papers [35]–[38]). Neither do we discuss the many sophisticated tools for text mining and natural language processing (e.g., [39]–[42]), for joining disparate concepts [43],[44], for literature-based discovery (e.g., [45]–[49], and for studies of bibliometrics [50],[51], literature dynamics [52], knowledge domains [53], detecting republication [54], and so on, all of which become considerably easier to implement only when all the necessary data are digitized and linked together with their relevant metadata.

**Figure 1:**
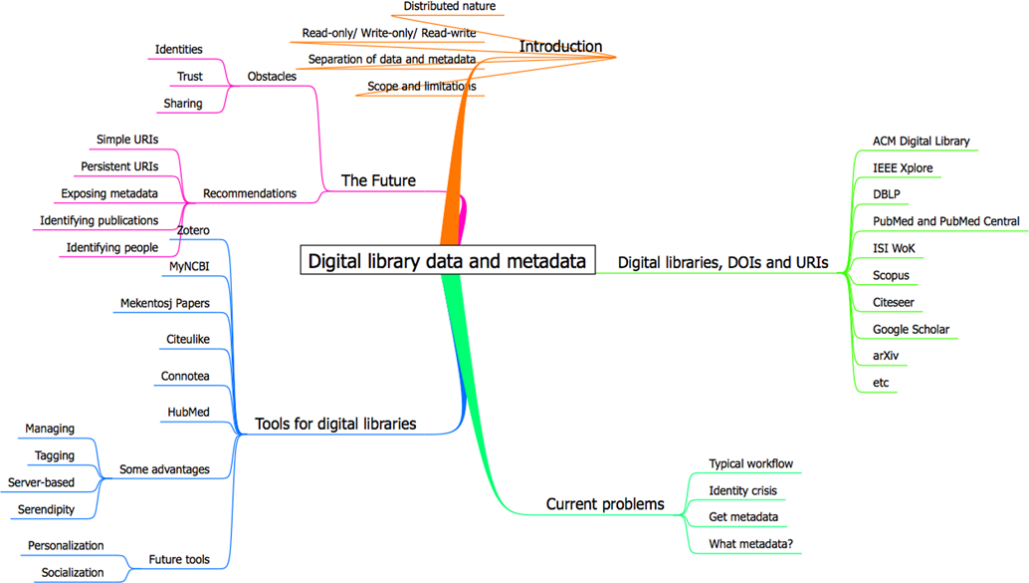
A mind map [207] summarizing the contents of this article in a convenient manner.

This Review is structured as follows (see also Figure 1): the section Digital Libraries, DOIs, and URIs starts by looking at the range of information in digital libraries, and how resources are identified using URIs on the Web. In the section Problems with Digital Libraries, we consider a fairly standard workflow that serves to highlight some problems with using these libraries. The following section, Some Tools for Defrosting Libraries, examines what Web-based tools are currently available to defrost the digital library and how they are making libraries more personal, sociable, and integrated places. Finally, the section A Future with Warmer Libraries looks at the obstacles to future progress, recommends some best practices for digital publishing, and draws conclusions.

## Digital Libraries, DOIs, and URIs

**Figure 2:**
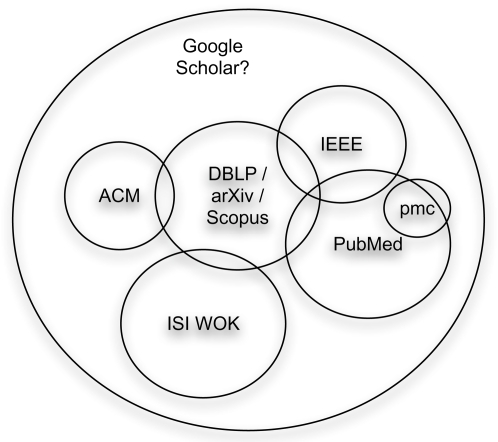
The approximate relative coverage and size of selected digital libraries described in the section Digital Libraries, DOIs, and URIs, and summarised in Table 1. Of all the libraries described, Google Scholar probably has the widest coverage. However, it is currently not clear exactly how much information Google indexes, what the criteria are for inclusion in the index, and whether it subsumes other digital libraries in the way shown in the figure. Note: the size of sets (circles) in this diagram is NOT proportional to their size, and DBLP, Scopus, and arXiv are shown as a single set for clarity rather than correctness.

Because computational biology is an interdisciplinary science, it draws on many different sources of data, information, and knowledge. Consequently, there exists a range of digital libraries on the Web identified by URIs [25] and/or DOIs [55],[56] that a typical user requires, each with its own speciality, classification, and culture, from computer science through to biomedical science. DOIs are a specific type of URI and similar to the International Standard Book Numbers (ISBN), allowing persistent and unique identification of a publication (or indeed part of a publication), independently of its location. The range of libraries currently available on the Web is described below, starting with those that focus on specific disciplines (such as ACM, IEEE, and PubMed) through to libraries covering a broader range of scientific disciplines, such as ISI WOK and Google Scholar. For each library, we describe the size, coverage, and style of metadata used (summarized in Table 1 and Figure 2). Where available, DOIs can be used to retrieve metadata for a given publication using a DOI resolver such as CrossRef [57], a linking system developed by a consortium of publishers. We illustrate with specific examples how URIs and DOIs are used by each library to identify, name, and locate resources, particularly individual publications and their author(s). We often take URIs for granted, but these humble strings are fundamental to the way the Web works [58] and how libraries can exploit it, so they are a crucial part of the cyberinfrastructure [59] required for e-science on the Web. It is easy to underestimate the value of simple URIs, which can be cited in publications, bookmarked, cut-and-pasted, e-mailed, posted in blogs, added to Web pages and wikis [60]–[62], and indexed by search engines. Simple URIs are a key part of the current Web (version 1.0) and one of the reasons for the Web's phenomenal success since appearing in 1990 [63]. As we shall demonstrate with examples, each digital library has its own style of URI for being linked to (inbound links) and alternative styles of URI for linking out (outbound links) to publisher sites. Some of these links are simple, others more complex, and this has important consequences for both human and programmatic access to the resources these URIs identify.

### 

#### The ACM Digital Library

The Association for Computing Machinery (ACM), probably best known for the Turing award, makes their digital library available on the Web [64]. The library currently contains more than 54,000 articles from 30 journals and 900 conference proceedings dating back to 1947, focusing primarily on computer science. Like many other large publishers, the ACM uses Digital Object Identifiers (DOI) to identify publications. So, for example, a publication on scientific workflows [65] from the 16th International World Wide Web Conference (WWW2007) is identified by the Digital Object Identifier DOI:10.1145/1242572.1242705. The last part of the DOI can be used in ACM-style URIs as follows: http://portal.acm.org/citation.cfmdoid1242572.1242705. Metadata for publications in the ACM digital library are available from URIs in the style above as EndNote [66] and BibTeX formats; the latter is used in the LaTeX document preparation system [67].

#### IEEE Xplore

The Institute of Electrical and Electronics Engineers (IEEE) provides access to its technical literature in electrical engineering, computer science, and electronics, through a service called Xplore [68]. The exact size of the Xplore archive is not currently described anywhere on the IEEE Web site. Xplore identifies publications using Digital Object Identifiers that are supplemented with a proprietary IEEE scheme for identifying publications. So, for example, a publication on text-mining [69] in *IEEE/ACM Transactions on Computational Biology and Bioinformatics* is identified by both the Digital Object Identifier DOI:10.1109/TBME.2007.906494 and an internal IEEE identifier 1416852. These identifiers can be used in URIs as follows: http://dx.doi.org/10.1109/TBME.2007.906494 and http://ieeexplore.ieee.org/search/wrapper.jsparnumber1416852. Metadata for publications in IEEE Xplore are available from URIs in the style above in EndNote, Procite, and Refman. Alternatively, publication metadata are available by using a DOI resolver such as CrossRef. Currently, the IEEE offers limited facilities for its registered members to build a personal library and to share this with other users.

#### DBLP

The Digital Bibliography and Library Project (DBLP) [70],[71], created by Michael Ley, provides an index of peer-reviewed publications in computer science. Recently, DBLP has started to index many popular journals with significant computational biology content such as *Bioinformatics* and *Nucleic Acids Research*, and currently indexes about 900,000 articles, with links out to full text, labeled EE for electronic edition. Thus an article by Russ Altman on building biological databases [72] is identified by the URI http://dblp.uni-trier.de/rec/bibtex/journals/bib/Altman04. Metadata for publications in DBLP are available in BibTeX format only. Unlike some libraries that we describe later, DBLP is built largely by hand [71], rather than by bots and crawlers indexing Web pages without human intervention. One of the consequences of this is that authors are disambiguated more accurately [73], e.g., where an author's middle initial(s) is not used or alternative first names appear in metadata. This kind of author disambiguation is particularly relevant to the naming conventions in some countries [74].

#### PubMed.gov and PubMed Central

PubMed [75] is a service provided by the National Center for Biotechnology Information (NCBI). The PubMed database includes more than 17 million citations from more than 19,600 life science journals [76],[77]. The primary mechanism for identifying publications in PubMed is the PubMed identifier (PMID); so, for example, an article describing NCBI resources [77] is identified by the URI http://pubmed.gov/18045790. Publication metadata for articles in PubMed are available in a wide variety of formats including MEDLINE flat-file format and XML, conforming to the NCBI Document Type Definition [77], a template for creating XML documents. PubMed can be personalized using the MyNCBI application, described later in the section Some Tools for Defrosting Libraries. PubMed Central [78], a subset of PubMed, provides free full-text of articles, but has lower coverage as shown in Figure 2. Related sites are also emerging in other countries, such as that in the UK [79]. A URI identifying the NCBI resources article [77] in the US PubMed Central is: http://www.pubmedcentral.nih.gov/articlerender.fcgiartid1781113. Metadata are available from URIs in PubMed Central as either XML, Dublin Core, and/or RDF [80] by using the Open Archives Initiative (OAI) [81] Protocol for Metadata Harvesting (PMH), a standard protocol for harvesting metadata. For example, embedded in the page identified by the URI above, there are Dublin Core terms such as DC.Contributor, DC.Date, and DC.title, which are standard predefined terms for describing publication metadata. In addition to such standard metadata, PubMed papers are tagged or indexed according to their MeSH (Medical Subject Heading) terms, curated manually.

#### ISI Web of Knowledge (WoK)

ISI WoK [82] is The Institute for Scientific Information's Web of Knowledge, a service provided by The Thomson Reuters Corporation, covering a broad range of scientific disciplines (not just computer science or biomedical science). The size of the library is somewhere in the region of 15,000,000 “objects” according to the footer displayed in pages of search results. Unfortunately, ISI WoK does not currently provide short, simple links to its content; so, for example, the URI for an NCBI publication [77] in ISI WoK is hidden behind a script interface called cgi [83]; this is usually displayed in the address bar of a Web browser, regardless of which publication is being viewed, as in this example: http://isiknowledge.com It is possible to extract individual URIs for publications, but regrettably they are usually too long and complicated and contain “session identifiers,” which make them expire after a set period of time (usually 24 hours). Temporary and long URIs of this kind cannot be easily used by humans, and prevent inbound links to the content. ISI WoK also provides various citation tracking and analytical features such as Journal Citation Reports, which measures the impact factor [84],[85] of individual journals [86]. Metadata for publications in ISI WoK are provided in BibTeX, Procite, Refman, and EndNote. WoK provides citation tracking features, particularly calculating the H-index [87] for a given author, as well as “citation alerts” that can automatically send e-mail when a given paper is newly cited.

#### Scopus.com

Scopus [88] is a service provided by Reed Elsevier and seems to be the Digital Library with individually the most comprehensive coverage, claiming (June 2008) >33,000,000 records (leaving aside Web pages). As far as linking is concerned, Scopus allows links <1?show=[to]?>to its content using OpenURL [89], which provides a standard syntax for creating URIs. For example, the URI http://www.scopus.com/scopus/openurl/document.urlissn03029743volume3298spage350 identifes a publication [90] from the Semantic Web conference, with the ISSN, volume, and page as part of the URI. The Scopus OpenURL link shown above is the simplest kind that can exist; many get much more complicated as more information is included in the URI, doubling the length of the one shown. The longer and more complicated URIs become, the less likely they are to be useful for humans. Scopus also links out to content using OpenURL and provides citation tracking. Metadata can be exported in RefWorks [91], RIS format (EndNote, ProCite, RefMan), and plain text, etc.

#### Citeseer

Citeseer [92] is a service currently funded by Microsoft Research, NASA, and the National Science Foundation (NSF), covering a broad range of scientific disciplines and more than 760,000 documents, according to Citeseer. The URI http://citeseer.ist.psu.edu/apweiler04uniprot.html identifies a paper about UniProt [93]. Publication metadata are available from Citeseer in BibTeX format, and citation tracking is performed annually in the Most Cited Authors feature [94].

#### Google Scholar

**Figure 3:**
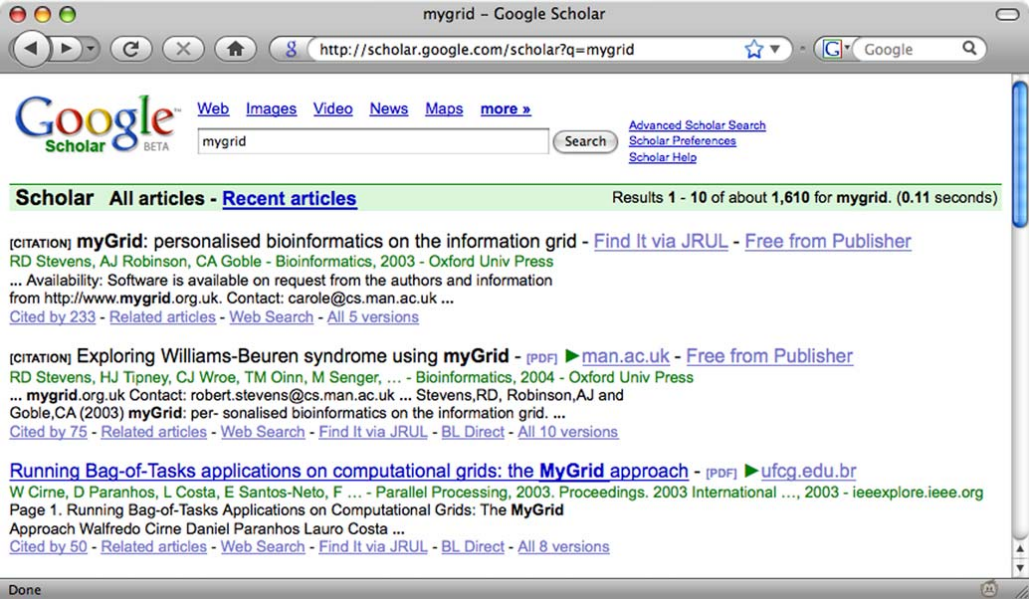
Google Scholar search results, identified by http://scholar.google.com/scholar?q = mygrid. Google Scholar links out to external content using a number of methods including OpenURL [89], shown here by the “Find it via JRUL” (JRUL is a local library) links. Unlike, e.g., WoK, it is relatively easy to create inbound links to individual authors and publications in Google Scholar; see text for details.

Google Scholar [95] (e.g., [96]–[99]) is a service provided by Google (see also [100]), which indexes traditional scientific literature, as well as preprints and “grey” self-archived publications [19] from selected institutional Web sites. A typical page from Google Scholar is shown in Figure 3. The size and coverage of Google Scholar does not seem to have been published, and the exact method for finding and ranking citations has not yet been made completely public [101].

In contrast to some other digital libraries, Google Scholar provides simple URIs that link to different resources. For example, http://scholar.google.com/scholarcites9856542662207029505 identifies citations of a publication [102] by Tom Oinn.

At the time of writing, Google Scholar does not currently offer any specific facilities for creating a personal collection of documents or sharing these collections with other users, other than using simple links such as the one above. Publication metadata can be obtained from Google Scholar where OpenURL links are found in its search results; otherwise, metadata can be obtained by clicking through the links to their original sources.

#### arXiv.org

arXiv [103] provides open access to more than 44,000 e-prints in physics, mathematics, computer science, quantitative biology, and statistics, and was created by Paul Ginsparg [104]. It is a leading example of what *can* be done, although it is presently little used by biologists. The arXiv has a different publishing model from that of the other digital libraries described in this paper, because publications are peer-reviewed *after* publication in the arXiv, rather than before publication. (A related but non-identical strategy is pursued with PLoS ONE, where papers are peer reviewed before being made accessible, but if they do not pass peer review they do not appear.) The arXiv is owned, operated, and funded by Cornell University and is also partially funded by the National Science Foundation. arXiv uses simple URIs to identify publications that incorporate the arXiv identifier. Because arXiv acts as a preprint server, some of its content eventually becomes available elsewhere in more traditional peer-reviewed journals. For example, an article on social networks published in *Science* [105] is also available from http://arxiv.org/abs/cond-mat/0205383. Metadata for publications in arXiv are available in BibTeX format, with various citation-tracking features provided by the experimental citebase project [106],[107]. This alternative approach to manual citation counts works by calculating the number of times an individual paper has been downloaded, as with the Highly-accessed feature on BioMedCentral journals.

#### …and the rest

In a short review such as this one, it is not possible to describe every single library a computational biologist might use, because there are so many. Also, it is surprisingly hard to define exactly what a specific digital library is because the distinction between publishers, libraries, and professional societies is not always a clean one. Thus, we have not described the digital libraries provided by Highwire [108], WorldCat [109], JSTOR, the British Library, the Association for the Advancement of Artificial Intelligence (AAAI), the Physical Review Online Archive (PROLA), and the American Chemical Society (ACS) (e.g., SciFinder). Neither do we discuss commercial publisher-only sites such as SpringerLink, Oxford University Press, ScienceDirect, Wiley-Blackwell, Academic Press, and so on here, since *most* of this content is accessible, typically via abstracts, via the other libraries and databases described in the section on digital libraries with links out to the publishers' sites.

#### Summary of libraries

Although they differ in size and coverage, all of these digital libraries provide similar basic facilities for searching and browsing publications. These features are well-documented elsewhere, so we will not describe them in detail here. With the exception of arXiv and PubMed Central, which provide full free access to entire articles, all other libraries described here provide free access to metadata (author, year, title, journal, abstract, etc.) and link to data (the full-text of a given article), which the user may or may not be licensed to view. The approximate relationship between the different libraries, as far as coverage is concerned, is shown in Figure 2.

Where these libraries differ is in the subscription, personalization, and citation-tracking features. So, for example, ISI WoK is a subscription-only service, not freely accessible, but which offers more extensive citation tracking features (such as ranking papers by citation counts, the impact factor [85],[86], and h-index [87]) than other libraries. Other services, such as the NCBI, are available freely, and provide additional features using custom tools to freely registered users. Other services such as Google Scholar and Citeseer are free, but currently offer no personalized view. Both ISI and Google Scholar provide services for counting and tracking citations of a given paper, which are not provided by most other libraries.

These libraries also differ considerably in the nature and power of their indexing by which users can search them on specific topics of metadata. Most permit Boolean searches on the basis of authors, keywords, words in a title or abstract, and so on, though none does this in real-time, and comparatively few allow sophisticated combinations.

All of this reflects the fact that these libraries and the means of searching them evolved independently and largely in isolation. Consequently, it is generally difficult for a user to build their own personalized view of *all* the digital libraries combined into one place, although tools described in the section Some Tools for Defrosting Libraries are now beginning to make this more feasible. Before we describe these further, we shall look at some of the current issues with using these digital libraries, as it is exactly these kinds of problems that have motivated the development of new tools. These tools, and the digital libraries they are built on, have to manage two inescapable facts: 1) redundancy: any given publication or author can be identified by many different URIs; 2) representing metadata: there are many different ways of identifying and describing metadata (and see Table 1). We describe some of the consequences of this in the next section.

## Problems Using Digital Libraries

**Figure 4:**
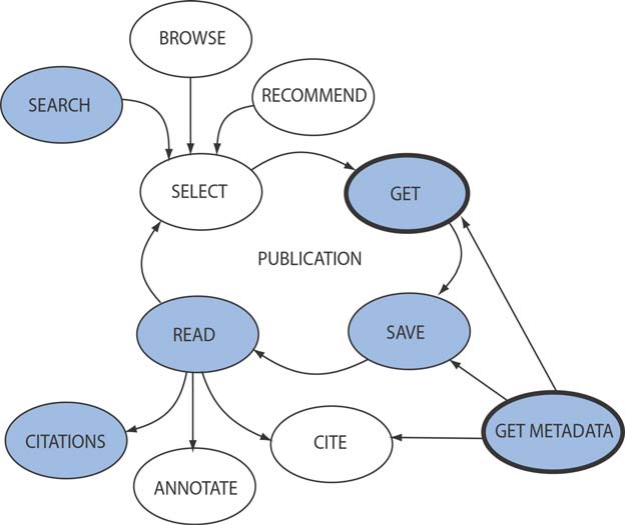
A typical workflow for using a digital library representing a subset of the literature. Tasks represented by white nodes are normally performed exclusively by humans, while tasks shown in blue nodes can be performed wholly or partly by machines of some kind. The main problematic tasks that make digital libraries difficult to use for both machines and humans are “GET” (publication) and “GET METADATA”. These are shown in bold and discussed further in the Identity Crisis section of this paper.

The digital libraries outlined in the previous section all differ in their coverage, access, and features, but the abstract process of using them is more standard. Figure 4 shows an abstract workflow for using any given digital library. We do not propose this as a universal model, which every user will follow, but provide it to illustrate some of the problems with managing data and metadata in the libraries described in the previous section on digital libraries.

To begin with, a user selects a paper, which will have come proximately from one of four sources: 1) searching some digital library, “SEARCH” in Figure 4; 2) browsing some digital library (“BROWSE”); 3) a personal recommendation, word-of-mouth from colleague, etc., (“RECOMMEND”); 4) referred to by reading another paper, and thus cited in its reference list (“READ”). Once a paper of interest is selected, the user: 1) retrieves the abstract and then the paper (i.e., the actual paper itself as a file), “GET” in Figure 4; 2) they save the paper, for example by bookmarking it, storing on a hard-drive, printing off, etc., (“SAVE”). Saving often involves getting the metadata, too, (“GET METADATA”). By metadata, we again mean the basic metadata about a publication, such as the author, date, journal, volume, page number, publisher, etc. In practice, this means any information typically found in an EndNote or BibTeX entry; 3) they read the paper, “READ” in Figure 4; 4) they may annotate the paper, (“ANNOTATE”); 5) finally, they may cite the paper (“CITE”). Citing requires retrieving the metadata, if these have not been retrieved already.

This abstract workflow is idealized, but highlights some problems with using current digital libraries, for both humans and machines. In particular, see the following list.

1. **Identity Crisis.** There is no universal method to retrieve a given paper, because there is no single way of identifying publications across all digital libraries on the Web. Although various identification schemes such as the PubMed identifier (PMID), Digital Object Identifier (DOI), ISBN, and many others, exist, there is not yet one identity system to “rule them all.”
2. **Get Metadata.** Publication metadata often gets “divorced” from the data it is about, and this forces users to manage each independently, a cumbersome and error-prone process. Most PDF files, for example, do not contain embedded metadata that can be easily extracted [110]. Likewise, for publications on the Web there is no universal method to retrieve metadata. For any given publication, it is not possible for a machine or human to retrieve metadata using a standard method. Instead there are many inadequate options to choose from, which add unnecessary complexity to obtaining accurate metadata.
3. **Which metadata?** There is no single way of representing metadata, and without adherence to common standards (which largely already exist, but in a plurality) there never will be. EndNote (RIS) and BibTeX are common, but again, neither format is used universally across all libraries.

We describe each of these issues more fully in the following sections.

### 

#### Identity crisis

We are suffering from an acute identity crisis in the life sciences [111]. Just as sequence databases have trouble managing the multiple identities of sequences [112], digital libraries also suffer from being unable to identify individual publications and their authors [113]. These are essential pieces of information that make libraries easy to use, and also help to track citations, but in the present implementation they create considerable barriers to users and machines. Any single publication or author is identified by numerous different URIs. An important task for managing these disparate collections involves reconciling and normalizing these different identity schemes, that is, calculating if two different URIs identify the same resource or not. For example, a human can fairly easily determine (by following the links) that each of these URIs identify the same publication, but writing a generic program to automate this for arbitrary URIs is more challenging: http://nar.oxfordjournals.org/cgi/content/full/36/suppl_1/D13; http://www.ncbi.nlm.nih.gov/pubmed/18045790; http://www.pubmedcentral.nih.gov/articlerender.fcgiartid1781113; and http://dx.doi.org/10.1093/nar/gkm1000.

Where DOIs exist, they are supposed to be the definitive URI. This kind of automated disambiguation, of publications and authors, is a common requirement for building better digital libraries. Unlike the traditional paper library, machines play a much more important role in managing information. They come in many forms, typically search-engine bots and spiders such as Googlebot [114], but also screen-scrapers [115], feed-readers [116],[117], workflows [102],[118], programs, Web services [90], [119]–[122], and ad hoc scripts, as well as semantic Web agents and reasoners [123]. They are obviously of great importance for text-mining [39]–[41], [124]–[126], where computer algorithms plus immense computing power can outperform human intelligence on at least some tasks [127]. Publication metadata are essential for machines and humans in many tasks, not just the disambiguation described above. Despite their importance, metadata can be frustratingly difficult to obtain.

#### Metadata: You can't always GET what you want

As well as the problem of extracting metadata from PDFs [110], getting metadata for any given URI which identifies a publication is also problematic. Although the semantic Web has been proposed as a general solution to this [128]–[132], it is currently a largely unrealised vision of the future [133],[134]. The Open Archives Initiative mentioned previously provides a solution to this problem, though it is not adopted by all publishers. So, given an arbitrary URI, there are only two guaranteed options for getting any metadata associated with it. Using http [135], it is possible to for a human (or machine) to do the following.

1. **http GET the URI.** Getting any URIs described in the previous section Digital Libraries, URIs, and DOIs will usually return the entire HTML representation of the resource. This then has to be scraped or parsed for metadata, which could appear anywhere in the file and in any format. This technique works, but is not particularly robust or scalable because every time the style of a particular Web site changes, the screen-scraper will probably break as well [136]. Some Web sites such as PubMed Central make this easier, by clearly identifying metadata in files, so they can easily be parsed by tools and machines.
2. **http HEAD the URI.** This returns metadata only, not the whole resource. These metadata will **not** include the author, journal, title, date, etc., of the publication but basic information such as the MIME type which indicates what the resource is (text, image, video, etc. [137]), Last-Modified date [135], and so on.

The lack of an adequate method for retrieving metadata has led to proposals such as the Life Sciences Identifier (LSID) [138],[139] and BioGUID [140] (Biological Globally Unique IDentifier). These may be useful in the future if they become more widely adopted, but do not change the current state of the digital library. As it stands, it is not possible to perform mundane and seemingly simple tasks such as, “get me all publications that fulfill some criteria and for which I have licensed access as PDF” to save locally, or “get me a specific publication and all those it immediately references”.

#### Which metadata?

Even if there were a standard way to retrieve metadata for publications, there is still the problem of how to represent and describe them. In addition to EndNote (RIS) and BibTeX, there are also various XML schemas such as the U.S. Library of Congress Metadata Object Description Schema (MODS) format [141] and RDF vocabularies, such as the Dublin Core mentioned earlier. Having all these different metadata standards would not be a problem if they could easily be converted to and from each other, a process known as “round-tripping”. However, some conversions gain or lose information along the way. Lossy and irreversible conversions create dead-ends for metadata, and many of these mappings are non-trivial, e.g., XML to RDF and back again [123]. In addition to basic metadata found in EndNote and BibTeX, there are also more complex metadata such as the inbound and outbound citations, related articles, and “supplementary” information.

The identity crisis, inability to get metadata easily, and proliferation of metadata standards are three of the main reasons that libraries are particularly difficult to use and search as automatically as one would wish. These are challenging problems to overcome, and the tools we describe in the next section tackle these problems in different ways.

## Some Tools for Defrosting Libraries

Although libraries can be cold, the tools described in this section could potentially make them much warmer. They do this in two main ways. **Personalization** allows users to say this is my library, the sources I am interested in, my collection of references, as well as literature I have authored or co-authored. **Socialization** allows users to share their personal collections and see who else is reading the same publications, including added information such as related papers with the same keyword (or “tag”) and what notes other people have written about a given publication. The ability to share data and metadata in this way is becoming increasingly important as more and more science is done by larger and more distributed teams [142] rather than by individuals. Such social bookmarking is already available on the Web site of publications such as the Proceedings of the National Academy of Sciences (http://www.pnas.org) and the journals published by Oxford University Press.

The result of personalization and socialization is integration of a kind that cannot be achieved by machines alone. First, we look at personalization-only style tools, then we examine tools that also allow socialization of the library through sharing.

### 

#### Zotero.org and Mendeley

Zotero [143] is an extension for the Firefox browser that enables users to manage references directly from the Web browser. As with most Web-based tools, Zotero can recognise and extract data and metadata from a range of different digital libraries. Users can bookmark publications, and then add their own personal tags and notes. Currently, Zotero does not allow users to share their tags in the same way that more “sociable” tools such as CiteULike and Connotea do (see below), although enhancements to the current 1.0 version of Zotero may include this feature. Zotero bookmarks cannot be identified using URIs, so it is not possible to link in from external sources to these personal collections. Mendeley [144] is a similar application that helps to manage and share research papers, although as well as having a Web-based browser version it is possible to store bibliographies using a more powerful desktop-based client that automatically extracts metadata from PDF files, but it can only do this where metadata is available in an amenable format [110].

#### MyNCBI

MyNCBI [77] allows users to save PubMed searches and to customize search results. It also features an option to update and e-mail search results automatically from saved searches. MyNCBI includes extra features for highlighting search terms, filtering search results, and setting LinkOut [145], document delivery, and external tool preferences. Like Zotero, MyNCBI currently allows personalization only, with no socialization features. It is also limited to publications in PubMed. As we have previously seen, computational biologists frequently require access to many publications outside PubMed, so they cannot capture their entire library in MyNCBI alone. Like Zotero, it is currently not possible to link to personal collections created in MyNCBI.

#### Mekentosj Papers

**Figure 5:**
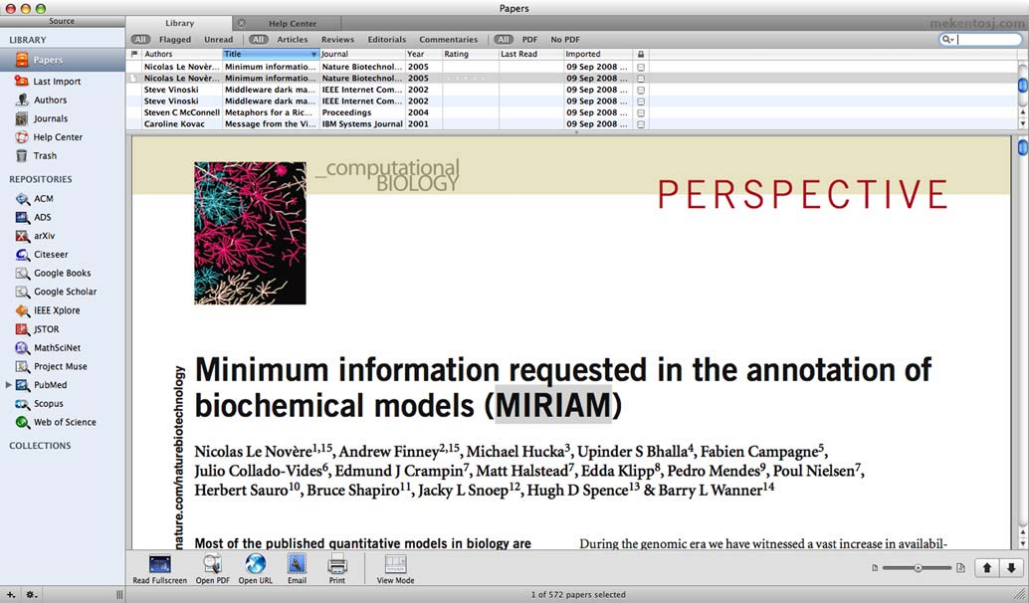
Mekentosj Papers can organize large collections of locally stored PDF files, with their metadata. It looks and feels much like the popular iTunes application, allowing users to manage their digital libraries by categories shown at the top. It is presently available only under Mac OS/X.

Papers [146],[147] is an application for managing electronic publications, originally designed by Alexander Griekspoor and Tom Groothuis. Although it is not a typical browser-based Web application, it can be closely integrated with several services on the Web-like Google Scholar, PubMed, ISI Web of Knowledge, and Scopus mentioned in the Digital Libraries section of this paper. The Papers application demonstrates how large collections of PDF files can be managed more easily. Papers provides a simple and intuitive interface shown in Figure 5 to a collection of PDF files stored on a personal hard drive. It looks and behaves much like Apple's iTunes, an application for managing music files, because the user does not have to know where the data (PDF file) is stored on their hard drive [110]. Unfortunately, Papers is only available for Apple Macintosh users, and there is no version for Windows, which limits its uptake by scientists.

The personalization of libraries is nothing especially new or groundbreaking, and scientists have been creating personal libraries for years, for example by having their own EndNote library or BibTeX file. Tools such as Zotero, MyNCBI, and Papers just make the process of personalization simpler. However, socialization of digital libraries *is* relatively new, in particular the ability of *multiple* users to associate arbitrary tags [27],[28],[148] with URIs that represent scientific publications. This is what CiteULike, Connotea, and HubMed (see below) all allow, thereby capturing some of the supposed “wisdom of crowds” [149] in classifying information.

#### CiteULike.org

CiteULike [150] is a free online service to organize academic publications, now run by Oversity. It has been on the Web since October 2004 when its originator was attached to the University of Manchester, and was the first Web-based social bookmarking tool designed specifically for the needs of scientists and scholars. In the style of other popular social bookmarking sites such as delicious.com [151],[152], it allows users to bookmark or “tag” URIs with personal metadata using a Web browser; these bookmarks can then be shared using simple links such as those shown below. The number of articles bookmarked in CiteULike is approaching 2 million, indicated by the roughly incremental numbering used. While the CiteULike software is not open source, part of the dataset it collects is currently in the public domain [153]. Publication URIs are simple: http://www.citeulike.org/article/1708098.

CiteULike normalizes bookmarks before adding them to its database, which means it calculates whether each URI bookmarked identifies an identical publication added by another user, with an equivalent URI. This is important for social tagging applications, because part of their value is the ability to see how many people (and who) have bookmarked a given publication. CiteULike also captures another important bibliometric, viz how many users have potentially *read* a publication, not just cited it. It seems likely that the number of readers considerably exceeds the number of citers [84],[150], and this can be valuable information. Time lags matter, too. This is particularly the case with Open Access, where the “most-accessed” *Journal of Biology* paper of 2007 [154] had in June 2008 been accessed in excess of 12,000 times, but has been cited just nine times (note that early access statistics can provide good predictors for later citations [155]). CiteULike provides metadata for all publications in RIS (EndNote) and BibTeX, providing a solution to the “Get Metadata” problem described in the previous section Metadata: You Can't Always GET What You Want, because every CiteULike URI for a publication has metadata associated with it in exactly the same way.

#### Connotea.org

Connotea [156] is run by Nature Publishing Group and provides a similar set of features to CiteULike with some differences. It has been available on the Web since November 2004. Connotea uses MD5 hashes [157] to store URIs that users bookmark, and normalizes them after adding them to its database, rather than before. This post-normalization means Connotea does not always currently recognize when different URIs (such as the examples in the section Identity Crisis) identify the same publication, a bug known as “buggotea” [158], which also affects CiteULike to a lesser extent. Like CiteULike, URIs in Connotea are simple. A publication about Connotea [156], for example, is identified by the URI http://www.connotea.org/uri/685b90ae66cfbc3fc8ebeed0a5def571. Metadata are available from Connotea in a wider variety of formats than from CiteULike, including RIS, BibTeX, MODS, Word 2007 bibliography, and RDF, but these have to be downloaded in bulk only, rather than individually per publication URI. The source code for Connotea [159] is available, and there is an API that allows software engineers to build extra functionality around Connnotea, for example the Entity Describer [160].

#### HubMed.org

HubMed [161] is a “rewired” version of PubMed, and provides an alternative interface with extra features, such as standard metadata and Web feeds [116],[117], which can be subscribed to using a feed reader. This allows users to subscribe to a particular journal and receive updates when new content (e.g., a new issue) becomes available. An example URI for a publication on HubMed [161] is http://www.hubmed.org/display.cgiuids16845111. Like CiteULike, HubMed also solves the “Get Metadata” problem because metadata are available from each HubMed URI in a wide variety of formats not offered by NCBI. This is one of HubMed's most useful features. At the time of writing, HubMed provides metadata in RIS (for EndNote), BibTeX, RDF, and MODS style XML. Users can also log in to HubMed to use various personalized features such as tagging.

#### Advantages of using CiteULike and Connotea

Both CiteULike and Connotea require users to invest time and effort learning how to use them, and importing or entering bibliographic information. Why should they bother? Managing bibliographic metadata using these tools has several advantages over the common scenario of storing un-indexed PDF files locally on a personal computer. Both CiteULike and Connotea provide a single place (a Web server) where data (PDFs) and metadata can both be shared and more tightly coupled; this has the following benefits.

##### Searching

Easier and more sophisticated searching is possible. Conversely, given a collection of PDFs on a hard drive, it is typically difficult (or impossible) to make simple queries such as “retrieve all papers by [a given author]”.

##### Managing

When authoring manuscripts, managing references in a Web-based repository can save some of the pain of re-typing metadata (e.g., author names) for a given publication. Provided the publication has a URI that is recognized by these tools, metadata are automatically harvested on behalf of the user, saving them time.

##### Tagging

Tags are just keywords, but these allow both personalisation and socialisation of bibliographic data, see [162] for papers cited in this Review as an example. Tagging of papers by other users allows non-expert users to explore related papers in ways that may not be possible through traditional reference lists, since exploring a subject of research in which you are not expert is made easier by following links added by other potentially more expert users.

##### Server-based

Hosting a bibliography on a Web server means that, if and when the user moves computer, the library is still accessible. However, keeping local and remote versions requires appropriate synchronisation, which can be problematic.

##### Serendipity

Many serendipitous discoveries [163] or intellectual linkages that may be determined via co-occurrences (e.g., [43], [49], [164]–[167]) exist in science, and these can be assisted by browsing links provided via social tagging.

#### Future tools

The tools described here are the first wave of Web 2.0, Library 2.0 [168], or even Science 2.0 [169] style tools that are helping to defrost the digital library. There will certainly be plenty more in the future; for example, the Research Information Centre [170] from the British Library is investigating innovative new tools in this area, backed by Microsoft. Some are calling it “Web 3.0” [171], but, whatever the name, it seems likely that we will see many digital library applications that will exploit the novel social features of platforms such as Facebook [172],[173] and OpenSocial [174]. Here they can exploit the identity mechanisms already built into those systems.

Personalization and socialization of information will increasingly blur the distinction between databases and journals [175], and this is especially true in computational biology where contributions are particularly of a digital nature. Scientific contributions to digital knowledge on the Web often do not fit into traditional scientific publishing models [31]. This is usually because they are either too “small” or too “big” to fit into journals. Web logs or “blogs” are beginning to fill the “too small” (see “microattribution” [176]) gap and can be used for communicating preliminary results, discussion, opinion, supplementary material, and short technical reports [177]–[179] in the style of a traditional laboratory notebook. Biological databases, such as those listed in the annual NAR database review [180], have long filled the “too big” gap in scientific publishing. They are clearly more significant than their publications alone. As we move in biology from a focus on hypothesis-driven to data-driven science [1],[181],[182], it is increasingly recognized that databases, software models, and instrumentation are the scientific output, rather than the conventional and more discursive descriptions of experiments and their results.

In the digital library, these size differences are becoming increasingly meaningless as data, information, and knowledge become more integrated, socialized, personalized, and accessible. Take Postgenomic [183], for example, which aggregates scientific blog posts from a wide variety of sources. These posts can contain commentary on peer-reviewed literature and links into primary database sources. Ultimately, this means that the boundaries between the different types of information and knowledge are continually blurring, and future tools seem likely to continue this trend.

## A Future with Warmer Libraries

The software described in the section Some Tools for Defrosting Libraries are a promising start to improving the digital library. They make data and metadata more integrated, personal, and sometimes more sociable. While they are a promising start, they face considerable obstacles to further success.

### 

#### Obstacles to warmer libraries

We suggest that the main obstacles to warmer libraries are primarily social [184] rather than technical in nature [185]. Identity, trust, and privacy are all potential stumbling blocks to better libraries in the future.

##### One identity to rule them all?

The basic ability to identify publications and their authors uniquely is currently a huge barrier to making digital libraries more personal, sociable, and integrated. The identity of people is a twofold problem because applications need to identify people as *users* in a system and as *authors* of publications. The lack of identity currently prevents answering very simple questions such as, ‘show me all person *x* publications’, unless the authors concerned are lucky enough to have unique names. Both the NCBI and CrossRef have initiatives to identify authors uniquely in digital libraries, but these have yet to be implemented successfully. The use of Single Sign-On (SSO) schemes such as Shibboleth [186] and OpenID [187] (the latter is used in projects such as myExperiment.org [188] and Connotea) could have a huge impact, enabling identity and personalization, without the need for hundreds of different usernames and password combinations. It remains to be seen what their impact on scientific literature will be. Technically, there are also tough challenges for creating unique author names [74],[113], such as synonymy, name changes, and variable use of initials and first names, which are ongoing legacy issues.

##### Who can scientists trust?

Passing valuable data and metadata onto a third party requires that users trust the organization providing the service. For large publishers such as Nature Publishing Group, responsible for Connotea, this is not necessarily a problem. That said, many users are liable to distrust commercial publishers when their business models may unilaterally change their data model, making the tools for accessing their data backwards incompatible, a common occurrence in bioinformatics. Smaller startup companies, who are often responsible for innovative new tools, may struggle to gain the trust of larger institutions and libraries. Most of the software described in the section Tools for Defrosting Libraries require a considerable initial investment from users to import their libraries into the system. Users have to trust service providers that this investment has a good chance of paying off in the longer term.

Scientists also have to decide how much to trust and rely on commercial for-profit companies to build and maintain the cyberinfrastructure they require for managing digital libraries. Not all commercial companies provide the best value-for-money services, and this is often true in scientific publishing. Paul Ginsparg, for example, has estimated that arXiv operates with a cost that is 100 to 1,000 times lower than a conventional peer-reviewed publishing system [189]. If the market will not provide scientists with the services they require, at a price they are willing to pay, they need to build and fund them themselves. The danger is that too much electronic infrastructure will be owned and run by private companies, and science will then be no better served than it was with paper-based publishing.

##### What data do scientists want to share?

Although the practice of sharing raw data immediately, as with Open Notebook Science [190], is gaining ground, many users are understandably cautious about sharing information online before peer-reviewed publication. Scientists can be highly secretive and reticent at times [191], selfishly not wanting to share their data and metadata freely with everyone and anyone, for fear of being “scooped” or copied without proper credit and attribution. Some tools provide security features, e.g., both CiteULike and Connotea allow users to hide references. However, this requires users to trust external providers to respect and protect their privacy, since the information is on a public server, and out of users' control.

## Recommendations

Warmer digital libraries cannot be achieved by software tools alone. The digital libraries themselves can take simple steps to make data and metadata more amenable to human and automated use, making their content more useful and useable. Only with proper and better access to linked data and metadata can the tools that computational biologists require be built. We make the following recommendations to achieve this goal.

### 

#### Simple URIs

URIs for human use should be as simple as possible, to allow easy linking to individual publications and their authors. Short URIs are much more likely to be used and cited [192] than longer, more complicated URIs.

#### Persistent URIs

It has been noted many times before [193],[194], but it is worth repeatedly restating: persistent URIs make digital libraries a much more useful and usable place. Although URIs will inevitably decay [195],[196], many (but not all) will be preserved by the Internet Archive [197],[198], and every effort should be made to keep them persistent where possible.

#### Exposing metadata

Publication metadata, in whatever style (EndNote, BibTeX, XML, RDF, etc.), should be transparently exposed and readily available, programmatically and manually, from URIs, HTML [199], and PDF files of publications.

#### Identifying publications

URNs (such as Digital Object Identifiers) should be used to identify publications wherever possible. Most large publishers already do this, although there are still many confounding exceptions.

#### Identifying people

This problem is twofold: people need to be identified as users of a system and as authors of publications. To tackle the first issue, tools and libraries should use Single Sign On (SSO) schemes, such as OpenID [187] to provide access to personalized features where possible, as this prevents the endless and frustrating proliferation of username/passwords to identify users in Web applications. The second requires unique author identification, an ongoing and as yet unsolved issue for digital libraries.

By following these recommendations, publishers, scientists, and libraries of all kinds can add significant value to the information they manage for the digital library.

## Conclusions

The future of digital libraries and the scientific publications they contain is uncertain. Rumours of the death of printed books [200] and the death of the journal [201] have (so far) been greatly exaggerated. In scientific publishing, we are beginning to see books and electronic journals becoming more integrated with databases, blogs, and other digital media on the Web. These and other changes could lead to a resurgence in the role of nonprofit professional societies and institutional libraries in the scientific enterprise [104] as the cost of publishing falls. But the outcome is still far from certain.

What is certain is the fact that we can look forward to a digital library that is more integrated, sociable, personalized, and accessible, although it may never be completely “frost-free”. Ultimately, better libraries will be a massive benefit to science. The current breed of Web-based tools we have described are facilitating this change, and future tools look set to continue this trend. Ultimately, data and metadata will become less isolated and rigid, moving more fluidly between applications on the Web. There are still issues with trust, privacy, and identity that may hinder the next generation of Web-based digital libraries, and these social problems will need addressing.

It has frequently been observed that scientists lag behind other communities in their use of the Web to communicate research [202], and that this is ironic given that the Web was invented in a scientific laboratory for use primarily by scientists [63]. Most scientists are painfully familiar with the shortcomings of the databases and software described in this Review, because these tools are at the very heart of science. Digital libraries are, and always will be, fundamental components of e-science, and of the “cyber-infrastructure” [59], [203]–[205], necessary for both computational and experimental biology in the 21st century.

**Box 1. Glossary and Abbreviations**

The following terms and abbreviations are used throughout this paper.

**API** Application Programming Interface. An API allows software engineers to re-use other people's software with standard programmatic “hooks.”

**Blog** WebLog, a suite of technologies for rapid publishing on the Web [177]–[179],[208],[209].

**DOI** Digital Object Identifier, a persistent and unique identifier for Objects, usually publications [55],[56], specific type of URN (see below and http://www.doi.org/).

**DTD** Document Type Definition, a template or schema for describing the structure of XML documents. The most prominent of these is that set down by the National Library of Medicine, http://dtd.nlm.nih.gov/, although each publisher tends to have their own.

**Dublin Core** A standard for describing metadata across many different domains, http://dublincore.org/.

**HTTP** Hypertext Transfer Protocol, a communications protocol used to transfer information on the Web [135].

**IETF** Internet Engineering Task Force develops and promotes Internet standards such as HTTP, URIs, http://www.ietf.org/.

**MeSH** Medical Subject Heading terms represent a controlled vocabulary used by the National Library of Medicine, http://www.nlm.nih.gov/mesh/.

**Metadata** Metadata are data about data, e.g., publication metadata include author, date, publisher, etc.

**MODS** Metadata Object Description Schema, a proposed standard for metadata emanating from the Library of Congress, http://www.loc.gov/standards/mods/.

**OpenURL** Standard syntax for URLs that link to scholarly publications, requiring an OpenURL resolver [89] to make use of them.

**OWL** Web Ontology Language, a W3C semantic Web standard for creating ontologies that makes extensive use of logical reasoners; see, e.g., [123],[210].

**RDF** Resource Description Framework, a W3C semantic Web standard for describing meta/data as graphs [123].

**SSO** Single Sign-On, a method for authenticating human users that allows one username/password to provide access to many different resources.

**URI** Uniform Resource Identifier, a URI can be further classified as a locator (URL), a name (URN), or both [25].

**URL** Uniform Resource Locator refers to the subset of URIs that, in addition to naming a resource, provides a means of locating the resource using, e.g., http://www.plos.org.

**URN** Uniform Resource Name, an identifier usually required to remain *globally unique* and *persistent*. Unlike URLs, URNs provide a mechanism for naming resources without specifying *where* they are located; for example, urn:isbn:0387484361 is a URN for a book, that says nothing about where the book can be located.

**W3C** The World Wide Web Consortium, http://www.w3.org/, an international standards body responsible for standards such as HTML, XML, RDF, and OWL, led by Tim Berners-Lee.

**Web 1.0** The original Web, the first version created in 1990 [63].

**Web 2.0** The Web in 2004, a phrase coined by Tim O'Reilly [26] to describe changes since 1990, such as “social software.”

**Web 3.0** Used to refer to future versions of the Web that do not yet exist [171]; for instance, (largely) the Semantic Web.

**Web feed** Web feeds allow users to subscribe to content that changes, and to be notified when it does, using either RSS or ATOM [116]. This can save time visiting Web sites manually to check for updates. Many journals now make Tables of Contents available in this way.

**XML** eXtensible Markup Language, a W3C standard for describing meta/data as “trees.”
